# An everlasting love: The relationship of happiness and meaning

**DOI:** 10.3389/fpsyg.2023.1046503

**Published:** 2023-03-13

**Authors:** Anastasia Besika

**Affiliations:** Department of Psychology, University of Zurich, Zürich, Switzerland

**Keywords:** happiness, meaning, psychological balance, well-being, values, consistency

## Abstract

*Happiness* is of great importance to people. Although *happiness* constitutes a central theme in psychology, the absence of a unifying theory and inconsistent terminology undermine scientific progress. The present article goes beyond attempting to define “types of happiness” or its contributing factors and addresses the role of *happiness* (i.e., embodied positive emotional patterns) as a function of a dynamic multisystem (i.e., an individual) and its relationship to *meaning* (i.e., ongoing bidirectional cognitive processes). As a dynamic multisystem, a person strives for stability as they move in physical space, and during their development, across time (i.e., dynamic balance). A primary requirement for dynamic balance is maintaining *consistency* by connecting the cognitive system to behavior. In psychological terms, such a connection is facilitated by meaning. The model suggests that happiness serves as a marker of a person’s consistency and meaningful interpretations of their lived experience. The model points to a new research direction.

## 1. Introduction

“What is the highest of all goals achievable by actions?… people...say it is happiness…but with regard to what happiness is they differ.”

*Aristotle (384 BC - 322 BC).*

“Man cannot stand a meaningless life”.

Jung, 1959.

*Happiness* that is associated with overall positive emotions and a sense of satisfaction, is central to human experience (Rokeach, 1973; Alexander et al., 2021). Although there has been significant progress over the last 40 years in understanding the conditions that contribute to making people happy, a fundamental lack of clarity over what defines *happiness* and what researchers measure (Schimmel, 2013) remains. The philosophical term *eudaimonia* refers to a different type of happiness to *hedonia* (i.e., experiencing pleasure) and is associated with experiencing *meaning* (Waterman, 2022). *Meaning* or *meaning in life* is conceptualized as ongoing cognitive processes (Heine et al., 2006) comprising comprehension (i.e., making sense of experiences), purpose (i.e., personal goals), and mattering (i.e., having a sense of personal importance) (George and Park, 2017).

The present article places happiness and meaning within a coherent framework that seeks to provide a speculative insight into the nature of interaction between emotion, cognition, and behavior. Drawing on poignant findings across many fields of psychology and beyond, the model seeks to explain the relationship between *happiness* and *meaning* as primary functions of a dynamic multisystem (i.e., an individual) that strives to balance. Maintaining *psychological balance* (i.e., alignment between cognition, emotion and behavior) requires *consistency*, which refers to the alignment between a person’s behavior (i.e., response to external situations and to others) and their cognitive patterns (i.e., self-concept, beliefs, motivational orientation, values and goals) (Besika et al., 2021). The proposed model suggests that *happiness*, as the experience of embodied positive emotional patterns, serves as a marker of consistency and is facilitated by *meaning* (i.e., ongoing bidirectional cognitive processes that connect cognition, emotion and behavior). Meaning allows a person to make sense of their environment and assign personal relevance to their experience in line with their cognitive patterns (e.g., goals) and informed by their experience, adjust their cognitive patterns. The bidirectional movement of meaning makes an experience meaningful and generates positive emotions (King and Hicks, 2021). In contrast, negative emotions mark inconsistency and indicate low levels or absence of meaning movement between cognitive patterns and behavior. In a state of inconsistency, a person struggles to make sense of personal experience, which may be perceived as meaningless. Negative emotional patterns may serve to activate adaptive re-adjustments in behavior and/or cognition to restore consistency and balance (Higgins, 1987; Brandtstädter and Greve, 1994).

**Figure 1:**
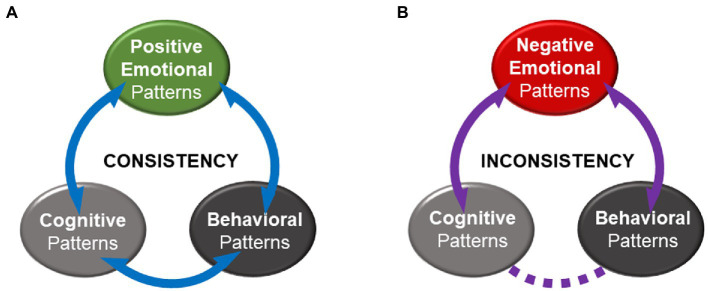
A model of Balance. **(A)** Positive emotional patterns indicate consistency, where meaning (i.e., ongoing bidirectional cognitive processes) connects behavior to cognition and emotion. **(B)** Negative emotional patterns indicate inconsistency, where there is interruption of meaning between cognition and behavior. Solid purple lines denote ongoing bidirectional cognitive processes that generate meaning. Dashed purple lines denote interruption in these processes.

The first section of this article presents a conceptual argument in support of the view that distinguishing different types of happiness (i.e., *eudaimonia* and *hedonia*) presents a barrier in understanding its nature. The argument demonstrates that any comparison between *eudaimonia* and *hedonia* is a false dichotomy, as the two philosophical concepts are unrelated, and further obscures the investigation of happiness with conceptual and methodological ambiguities (Kashdan et al., 2008). Drawing on the Aristotelian idea that *balance* is the key to *happiness* and adopting a system dynamics perspective, the second section presents a theoretical model that explains *happiness* (i.e., embodied positive emotional patterns) and *meaning* (i.e., ongoing bidirectional cognitive processes) as primary functions of a dynamic multisystem (i.e., an individual). Meaning facilitates the alignment between cognitive, emotional and behavioral patterns (i.e., psychological balance; Besika et al., 2021), whereas happiness serves to signal their level of alignment (Figures 1A,B). Altogether, this work addresses the overarching question of how emotion and cognition contribute to maintaining well-being. In line with the definition of WHO (World Health Organization, 2021), well-being refers to a subjective positive state that includes the ability to contribute to the world with a sense of meaning and purpose. In this context, happiness and meaning are functional abilities that enable well-being.

### 1.1. Hedonia vs. Eudaimonia: A false dichotomy

In a large part of the literature, happiness is used interchangeably with the term *Subjective Well-Being* (SWB; Diener, 2009) (i.e., subjective affective and cognitive evaluations of life), as well as with a range of other terms including *psychological well-being* (Ryff et al., 1995), *wellness* (Cowen, 1991), *authentic happiness* (Seligman, 2002) or *positive orientation* (Oleś and Jankowski, 2018). Introducing *eudaimonia* in psychology research created a conceptual discrepancy since this broad concept did not match the prevailing conceptions of happiness. Waterman (2022) suggests that Ross (1956) wrongly translated *eudaimonia* into happiness in Aristotle’s *The Nicomachean Ethics* and draws a sharp conceptual distinction between the two that reduces happiness to *hedonia* (i.e., seeking pleasure) and associating *eudaimonia* with meaning and optimal functioning (Waterman, 2022). These conceptual re-adjustments introduce a new psychological construct and overlook the philosophical background of the terms *hedonia* and *eudaimonia*.

#### 1.1.1. Definitions and etymology

The dictionary of Modern Greek (Petrounias, 2018) defines eudaimonia, as a blissful state resulting from a continuous effort for moral perfection and achieving self-actualization, which can be evaluated at the end of one’s life by others. This definition is in line with Aristotle’s (384–322 BC) concept that refers to a normative way of living concerning the “activity of the soul in accordance with virtue” (Ross, 1956, Book 11, p. 12). In spite of the etymological interpretation of eudaimonia (i.e., “eu = good and daimon = demon”) not making psychological sense (Kashdan et al., 2008), psychologists interpret eudaimonia as a type of happiness that involves subjective experiences of meaning (e.g., Vittersø, 2016). Given the absence of an equivalent English word, Ross’s translation of eudaimonia into happiness served its purpose. However, considering the theory eudaimonia represents, self-reports are not appropriate for assessing the normative question of what makes life virtuous or the degree of a person’s virtuousness. Should psychologist insist on using a Greek term for operationalizing happiness, eftehea (ευτυχία) is a more appropriate term, translating into a state of physical and mental satisfaction that derives from the achievement of goals (Petrounias, 2018), which is in line with the widely used term SWB (Diener, 2009). However, the plethora of terms and definitions generate confusion that undermines the scientific nature of studying outcomes as orphans of a comprehensive theoretical framework.

#### 1.1.2. Philosophical background of hedonia and eudaimonia

Any comparison between *eudaimonia* and *hedonia* (e.g., Huta, 2018) as different types of happiness is a false dichotomy that seems to be rooted in misinterpretations of the teachings of Aristippus (435–356 BC) and Aristotle (384–322 BC). The two ancient philosophers set objective standards for living well. Aristippus promotes *hedonia* and striving to achieve the highest degree of physical pleasure and the satisfaction of basic instincts, at any cost: “Pleasure is the sole good...and…only one’s own physical, positive, momentary pleasure is good, and is so regardless of its cause” (Tatarkiewicz, 1976, p. 317). In contrast, Aristotle who promotes *eudaimonia* as the ultimate good in life, considers eudaimonious a person “who is active in accordance with complete virtue and sufficiently equipped with external goods, not for some change period but throughout a complete life” (Ross, 1956, Book 10, p.16). The above quotes encapsulate the normative nature of the two philosophical teachings, which are concerned with the kind of life people should lead and not with their subjective experiences. Aristippus, who suggests setting hedonia as a top priority, speaks about pleasure and not about happiness. Aristotle promotes nurturing a good spirit as the “the ultimate good in life.” Whether maximizing pleasure at any cost is better than striving for soul purification is a matter of personal choice. One may argue that both theories constitute extreme approaches to life. However, such argument is beyond the scope of this article, which merely aims to emphasize that both philosophers make normative claims regarding standards of living and not regarding the subjective experience of happiness. In addition to methodological shortcomings (Kashdan et al., 2008), attempting to evaluate matters of the soul using self-reports is conceptually inappropriate and practically infeasible.

### 1.2. Happiness and meaning: An everlasting love

Happiness is a very complex concept (Kringelbach and Berridge, 2010) and research identifies a non-exhaustive list of factors associated with it, demonstrating the complexity of what it may entail. For example, physical factors (e.g., genetics; McCourt et al., 1999), personality traits (e.g., extraversion, neuroticism and temperament; Cowan, 2019), demographics (e.g., relationship status, gender, income, health and education; Lomas and VanderWeele, 2023), satisfaction of basic psychological needs (Feng and Zhong, 2021), social relationships (Bai et al., 2021), time perspective and forgiveness (Allemand et al., 2012), and many more. Research shows that meaning is strongly associated with happiness (Karataş et al., 2021) and it overlaps with hedonic pleasure (Huta, 2018). Evidence confirms that happiness may involve both meaningful and hedonic experiences, as people typically evaluate their happy experiences as meaningful (King and Hicks, 2021). Studies replicating Nozick’s (1974) thought experiment test the hypothesis that people prefer to derive happiness from experiences that are meaningful (Hindriks and Douven, 2018). Participants chose among three hypothetical scenarios that would make them feel happy: (a) disconnecting from reality and connecting to a machine that simulates pleasant experiences, (b) taking a pill that induces pleasure, and (c) taking a pill that enhances functionality while remaining in touch with reality. The majority of participants preferred to take the pill that enhances daily functionality while remaining connected to reality. These results indicate that the need for engagement with the external world precedes the need for experiencing pleasure.

Neuroscience findings support the idea that the presence of meaning facilitates a person’s connection to the external world. Meaning provides a sense of coherence that contributes to feeling safe and perceiving the world as predictable and controllable (Davis and Panksepp, 2011). Meaning is associated with a range of psychological benefits including, ability to cope better with adversity (Rose et al., 2023); enhancement of health and stress moderation (Schnell and Krampe, 2020); work enjoyment (Bonebright et al., 2000); high levels of self-esteem (Lew et al., 2020) and life satisfaction (Wolfram, 2022). In contrast, low levels of meaning are associated with a range of negative outcomes such as substance misuse (Csabonyi and Phillips, 2020), stress (Trzebiński et al., 2020), and suicidal ideation (Marco et al., 2020). When one feels depressed, it is difficult to experience meaning (King et al., 2006). In contrast, experiencing positive emotions and pleasure enhances the perception of life as meaningful, which in turn fosters happiness (King and Hicks, 2021). Life satisfaction arises from the coexistence of pleasurable and meaningful activities, such as personal involvement and personal expression (King and Hicks, 2021). In addition, meaningful interpretations of events generate positive affect (Reker and Wong, 1988; Wong et al., 2021) and over time such interpretations can establish a conceptual link between experience and emotion, forming the idea that life is meaningful (Clore and Palmer, 2009).

The plethora of research findings indicates an *everlasting love* between happiness and meaning; a reciprocal relationship where meaning (e.g., a sense of purpose, coherence and mattering) cannot exist without positive emotion and positive emotion cannot exist without meaning. Thus, distinguishing them as different routes to happiness (e.g., Huta, 2020; Waterman, 2022) presents a barrier in forming a unifying theoretical framework. In addition, maintaining a research focus on *what* makes people happy and *what* they find meaningful diverts research from investigating the universal mechanism that facilitates subjective experiences of happiness and meaning. Focusing on the functionality of happiness and meaning instead, and on their relationship can help understand *how* a person maintains well-being. Addressing questions such as, *how* do cognitive patterns interact with the environment, and *how* does the environmental context influence such interactions may lead to a nomothetic model that explains the functionality of happiness and meaning. It is inevitable that such an investigation will involve more than two or three variables (Sanbonmatsu and Johnston, 2019).

## 2. Toward a unifying theoretical framework

Despite the volume of references to Aristotle and suggestions that *balance* can serve as a guide in developing accounts of happiness (Grant and Schwartz, 2011), psychologists paid scant attention to Aristotle’s “golden mean.” Typically, “balance is used to describe the relationship quality between two dialectically related phenomena” (Lomas, 2021, p. 51). Although previous models acknowledge that balance is a unifying principle that pertains to all domains of well-being and “constitutes a cluster of conceptually related dynamics” (Lomas, 2021, p. 50), a model is yet to explain how a person maintains balance as an integral organism across all its levels of functioning (i.e., cognition, emotion, behavior). A combination of social psychology findings and of principles that govern dynamic systems leads to the hypothesis that the “golden mean” is a multifaceted psychological state and a prerequisite for happiness. Unlike previous models that provide a narrative categorization of the different contexts within which obtaining balance is important (e.g., Lomas, 2021), a parsimonious model explains that *consistency* across a person’s multiple levels of functioning is a primary condition for maintaining psychological balance (Besika et al., 2021).

As a person comprises of multiple systems such as cognitive, emotional and physical that are subject to change over time, an individual constitutes a dynamic multisystem (Perone et al., 2021). The primary function of a dynamic system is its stability, which requires the alignment between the system’s structure and its behavior (Schöner and Kelso, 1988; Ford, 1999). Drawing on the *Action Identification Theory* (Vallacher and Wegner, 1987) the model assumes that as well as a physical structure (e.g., body), a person’s cognitive system (e.g., motivational, value and goal patterns) has a hierarchical structure that drives their behavior. *Consistency* (i.e., alignment between cognition and behavior) is identified as a primary requirement for an individual to balance psychologically (Besika et al., 2021). Meaning (i.e., ongoing bidirectional cognitive processes) is considered to facilitate consistency as it connects a person to their external environmental context by receiving feedback that informs their behavior, which in turn influences their cognitive system. Meaning and consistency are associated with happiness (Mason et al., 2019; King and Hicks, 2021). Being consistent, a person’s cognitive patterns (i.e., their self-concept, beliefs, motivational orientation, values and goals), are aligned to their behavior. In such a state, an individual experiences overall positive emotions. In a state of inconsistency, a person’s cognition is not aligned to their behavior and they experience overall negative emotions. Happiness or experiencing positive emotions indicates the presence of meaning that connects an individual to their environmental context (see Figure 1A), whereas negative emotions indicate an interruption in meaning and a disconnection between cognition and behavior (see Figure 1B). Thus, happiness, as embodied positive emotional patterns, signals consistency within the person. Heightened negative emotions serve to activate cognitive processes that generate meaning, which may lead to re-adjustments in cognitive patterns and/or behavioral patterns in aiming to restore consistency (Higgins, 1987). In this state of balance, a person perceives their experience as meaningful as they can relate to it (see Figure 1A).

### 2.1. Dynamic balance of a multisystem

#### 2.1.1. Physical, cognitive, and emotional interactions

Research demonstrates dynamic interactions between a person’s physical (i.e., biological), cognitive and behavioral levels of functioning. For example, biology research indicates that genotypes (i.e., the genetic makeup of human body) moderate children’s sensitivity to maltreatment and the possibilities of developing antisocial behavior (Caspi et al., 2002). Moreover, research findings show that cognitive processes contribute to adapting physical movement to the environment and maintaining physical balance (Teasdale et al., 1993). These findings indicate that the system’s balance relies on interactions between biological and cognitive processes.

Neuroscience findings confirm ongoing interactions between emotion and cognition. Research has long established that neurological circuits generate fleeting pleasure through sensory satisfaction (MacLean, 1978). Primary, pleasure emerges from emotion-generating circuits, whereas cognitive pleasure is generated by secondary brain activity, such as thoughts about how internal and external states relate. Complex processes arise from cognitive awareness regarding emotional states (Panksepp, 2003, 2005). Areas in the pre-frontal cortex of the brain enable higher levels of regulatory control and endow a person with the ability to form goals and abstract concepts, such as values and future planning (Rushworth et al., 2011). Moreover, neurobiological mechanisms, which are responsible for producing sensory pleasures, are involved in producing pleasure through activity engagement. For example, a meta-analysis of studies based on functional magnetic resonance imaging (fMRI) explains that sexual desire (i.e., physical pleasure) and love (e.g., emotional association) are both mental states of intense longing for union with others. The neural circuits that produce both bodily pleasure and love share a common set of brain areas (Cacioppo et al., 2012). Thus, it would be impossible to experience happiness without genetically encoded neural structures (Panksepp and Watt, 2011). In addition, happiness requires evaluation of actions and goals in relation to their mental representations (Davis and Panksepp, 2011).

It is a common understanding that people operate in a physical as well as in a cognitive environment and strive to adapt their behavior to changes that may occur both in physical space (e.g., situational changes) and across time (e.g., aging). The ability to adapt to spatio-temporal changes makes an individual a dynamic system that operates on many levels (e.g., physical, cognitive, emotional). Primarily, as a person moves in space and time they need to maintain physical balance, which requires physical *consistency* (i.e., alignment and coordination between the body parts) and physical *flexibility* (i.e., dynamic re-adjustments in the organization of the body parts) (Horak, 2006; Kwon et al., 2013). Drawing on the principles that apply to dynamic systems, the proposed model postulates that the primary functional requirement to maintain balance is equally relevant to a person’s psychological functioning. In this regard, a person achieves psychological *consistency* through the alignment and coordination between their cognitive patterns (e.g., values) and their behavior (Besika et al., 2021). In addition, psychological *flexibility* (i.e., dynamic re-adjustments in the internal organization of either cognitive components or/and in behavior patterns) may rely on bidirectional ongoing cognitive processes that connect cognition to behavior and help a person make sense of their experience and the outside world.

#### 2.1.2. Finding the “Golden mean”

##### 2.1.2.1. Virtues are socially predefined

In the Nicomachean Ethics (Ross, 1956) Aristotle emphasizes that balance is the key to happiness and conceptualizes a virtue as the “golden mean” between excess and deficiency. For example, the virtue of *being friendly* is the “golden mean” of being slavish and being cranky. Each virtue is bound by the individual’s capacity and their situational context, and it is not the mid-point in an *excess - deficiency* continuum. Aristotle names approximately 18 virtues and suggests manifesting as many virtues as possible, not in isolation but rather as an overall behavior that displays compassion toward others, may increase ability to function well. Therefore, striking a balance may entail a multifaceted “golden mean,” or a cognitive pattern of values that may inform behavior.

Whereas Aristotle’s virtues reflect ideals of his social context (e.g., magnificence), social psychology research identifies a set of universal values representing the current socially predefined virtues. Multicultural studies show that a set of value domains (i.e., Security, Power, Achievement, Hedonism, Stimulation, Self-Direction, Universalism, Benevolence, Conformity, and Tradition) serve as guiding principles within the social framework of all cultures and represent ideals that influence people’s behavior (Schwartz, 1992; Schwartz and Cieciuch, 2021). Through socialization processes that occur within different settings (e.g., education, family, work), individuals integrate universal ideals to a different degree (Cieciuch et al., 2016; Besika, 2022). Recent evidence reveals that people cognitively integrate a shared pattern of universal values, with Power being at the lowest boundary and Benevolence forming the highest boundary of the pattern. The overall level of integration of this value pattern provides meaningful information regarding people’s level of well-being. People with a high level of value orientation report higher levels of meaning and life satisfaction than those with a lower level of value orientation (Besika, 2022). Those with a higher level of association between their values and daily experiences report higher average levels of meaning and life satisfaction compared to those with a low level of association (Besika et al., 2022). These findings reflect earlier research that shows that not sharing group values is associated with overall low levels of well-being and physical health (Dressler and Bindon, 2000).

The above evidence supports the hypothesis that alignment between cognitive patterns (e.g., value pattern) and behavior is pertinent to happiness and well-being.

##### 2.1.2.2. Virtues within a dynamic system

In line with the principle of bifurcation (i.e., division into parts) that governs dynamic systems (Arnold et al., 2013), beyond certain points of either excess or deficiency the value pattern may change from a pattern of virtues to a pattern of vices (Grant and Schwartz, 2011). Under certain conditions a dynamic system loses its coherence and degrades into a chaotic state and “...the slightest disturbance in the psychological as well as in the biological equilibrium may be detrimental...” (Jung, 1977, p. 451). As the present article is concerned with the primary psychological conditions that facilitate happiness, it is outside its scope to investigate the conditions under which a person’s value patterns start becoming a threat to their psychological balance and happiness.

### 2.2. Happiness and meaning as functions of a dynamic multisystem

This section investigates the environmental contexts of an individual in aiming to explain the functionality of happiness and meaning within a dynamic multisystem (i.e., a person). A person’s physical body has a universal structure (Hernandez et al., 2018). Is there a universal cognitive structure? An integration of psychology findings reveals that ongoing cognitive processes that facilitate psychological consistency by connecting a person’s external to their internal environmental contexts, lead to the formation of primary cognitive components with a hierarchic structure. What is the role of emotion and how does it relate to these cognitive processes? Addressing such questions requires integrating knowledge from many domains (Schimmack, 2008).

#### 2.2.1. The cognitive environment: A universal structure

##### 2.2.1.1. Primary cognitive components

Throughout development, people are constantly engaged in making sense of their external environment and of themselves. Ongoing cognitive processes that generate meaning help them construct an identity in line with their social context and culture (Zittoun and Brinkmann, 2012). An individual constructs the primary cognitive component of a *self-concept* (i.e., mental self-representations in relation to the past, present and future) (Brandtstädter and Greve, 1994). As people continuously compare their self-perceptions of who they wish to be, who they ought to be and who they actually are with others’ perceptions of them they construct *self* and *others* representations within their self-concept (Higgins, 1987). The *value pattern* constitutes another primary cognitive component that represents a person’s social context (Rokeach, 1973). Longitudinal studies show that throughout their development, people integrate universal values at a different level of importance (Cieciuch et al., 2016; Coelho et al., 2019; Besika, 2022). Studies indicate that a person’s values are characterized by an interest to either serve the *self* and/or *others.* In addition, a pattern of four motivational orientations (i.e., self-enhancement, conservation, self-transcendence, openness-to-change) underlies a person’s values. Altogether, these cognitive components inform personal goals and influence behavior (e.g., Sortheix and Schwartz, 2017).

##### 2.2.1.2. The organization

Through ongoing bidirectional cognitive processes (i.e., meaning-making processes) that encode the physical environment into symbols of personal significance, and in turn decode these symbols into meaningful information and experiences (Heine et al., 2006), a person develops primary cognitive components that represent their physical context. Research indicates that the cognitive components have a vertical hierarchy: (1) The *self-concept*, a cognitive pattern denoting the relationship of a person with themselves and with other people (Higgins, 1987). (2) The *motivational orientation pattern*, denoting ways an individual may perceive their relationship to the external world (i.e., conservation, openness to change, self-enhancement, and self-transcendence) (Schwartz, 1992). (3) The *value pattern*, denoting the way a person perceives the ideals of their socio-cultural context (Schwartz, 1992). (4) The *goal pattern*, denoting a person’s desired end-states that influences behavior (Vallacher and Wegner, 1987). Accordingly, behavior is meaningful when it serves a higher order goal. In line with the hypothesis that Aristotle’s “golden mean” is multifaceted, additional studies indicate that the cognitive environment has a horizontal dimension and that an increased capacity for operating in multiple domains is positively associated with well-being (e.g., Marks and MacDermid, 2006).

#### 2.2.2. Adaptive re-adjustments restore happiness

As any other dynamic system (Ford, 1999) a person needs to satisfy the requirements imposed by the law of dynamic balance (Kwon et al., 2013), which requires that the system maintains equilibrium by being consistent and flexible (Horak, 2006). The literature supports the idea that maintaining psychological balance requires consistency between a person’s cognitive and physical environments and adaptive re-adjustments in response to change (e.g., Besika et al., 2021). Experimental research indicates that dynamic re-adjustments occur within the cognitive pattern components. For example, the dual pattern of *self* (i.e., the individual) and *others* (i.e., significant others or generally others) fluctuates systematically in response to change. When a situation requires placing more focus on the *self*, people shift their focus from *others* and vice versa (Gaertner et al., 2008). Moreover, longitudinal experiments report that a person’s values behave as a dynamic system as they fluctuate systematically in response to life events. Increase of importance in one value follows decrease of importance in another and the degree of fluctuation positively correlates with the severity of the event (Bardi et al., 2009).

Typically, *self* and *others* are perceived as opposing ideas that generate cognitive dissonance. The suggestion that an increased capacity to tolerate cognitive dissonance could increase cognitive and emotional maturity (e.g., Wong et al., 2021) somehow conflicts with the idea of well-being. In contrast, the proposed model views the two mental representations of *self* and *others,* as complimentary cognitive patterns that their dynamic interaction facilitates adaptation to change. Adaptive re-adjustments may involve shifting importance from *self* to *others* or vice versa, (Gaertner et al., 2008), which may inform changes in value priorities (Bardi et al., 2009) and result in re-defining meaningful goals and/or changing behavior (Brandtstädter and Greve, 1994). For example, *John* who deeply cares for his family (e.g., prioritizes the value of *family*) and is committed to looking after them may decide to go on holiday as he finds himself feeling very tired. Hence, *John* shifts his focus from *others* to *self* and prioritizes the value of *health* in response to changes in his physical behavior, as he needs to maintain consistency. Thus, fluctuations in the importance a person places on either *self* or *others* in response to what the situation demands aim to maintain consistency across the different levels of functioning. Consistency across cognitive components (e.g., values and goals) is associated with *happiness* and *meaning* (Besika et al., 2021). In contrast, inconsistencies between the cognitive and physical environmental contexts of a person generate intense negative emotions, which may activate adaptive re-adjustments (Clore and Schnall, 2005; Clore and Ortony, 2008; Mason et al., 2019). Thus, the systemic behavior of the two seemingly contradictive mental contexts may facilitate dynamic re-adjustments that restore emotion.

#### 2.2.3. Meaning-making processes

Aristotle suggests that finding balance is possible in any situation. For example, when dealing with anger, balance requires being angry at the right time, with the right people and for the right reason (Ross, 1956, in the *Nicomachean Ethics* translation). This implies an alignment between emotion, cognition and behavior as well as an alignment across the person’s spatio-temporal context, which may include other people. Such an alignment relies on meaningful interpretation of events (Csikszentmihalyi, 1990), as meaning operates in the motivational, cognitive and affective levels of functioning (Reker and Wong, 1988). Bidirectional movement of meaning-making processes may lead a person to meaningful interpretations that allow them to make sense of a situation (Zittoun and Brinkmann, 2012) and translate emotion into information (King and Hicks, 2021). Hence, meaning facilitates psychological balance and fosters positive emotion (King et al., 2006; Heintzelman and King, 2014; Jamieson et al., 2018). Informed by the above, the proposed model assumes that meaning in the form of ongoing bidirectional cognitive processes a) provides information about emotion b) provides feedback regarding the impact of behavior on the environment, which includes other people, and c) facilitates comparisons between a person’s cognitive patterns and their behavior, which may lead to adaptive cognitive and/or behavioral re-adjustments.

#### 2.2.4. A negative feedback loop mechanism maintains emotional equilibrium

As change occurs in a person’s physical and/or cognitive environmental contexts, an individual faces the ongoing challenge of making adaptive re-adjustments. Which mechanism facilitates adaptation? The *control theory of self-regulation* (Carver and Scheier, 2019) explains that a negative feedback loop mechanism reduces discrepancies between a person’s cognitive states and physical environment as it aims to maintain a ‘set point’ of happiness (i.e., an individual homeostatic emotional equilibrium) (Heady and Wearing, 1992; Heady, 2006). Genetics mainly influence a person’s emotional equilibrium (McCourt et al., 1999) and homeostatic processes keep it relatively stable at the individual’s baseline (Heady, 2006). Meaning-making processes help a person make adaptive re-adjustments in response to change by providing feedback regarding their state of consistency and alignment to their physical environment. Detecting a mismatch generates emotional discomfort that may lead to cognitive and/or behavior re-adjustments. Processes within the negative feedback loop mechanism aim to restore emotion (e.g., Mason et al., 2019) and return a person to their ‘set point’ of happiness (Clore and Schnall, 2005; Heady, 2006; Heintzelman and King, 2014; Mason et al., 2019). Studies indicate that this “set point” is typically positive (Diener and Diener, 1996). Moreover, a study where multi-national participants (*N =* 2,392 and *N* = 6,239) ranked their ideal level of happiness on a continuum from 0 (*only sadness, no happiness ever)* to 100 (*only happiness, no sadness ever)* reports that the overall ratings do not exceed 80%. In the absence of all restrictions, people’s ideal level of happiness hovers just below 70% in collectivist cultures and just above 70% in individualistic cultures (Hornsey et al., 2018). Below a certain level of positive emotion people experience homeostatic failure, which is an indication that external life circumstances have control over the regulatory mechanism (Cummins, 2003). The above empirical evidence suggests that happiness serves as a marker of consistency, which ensures that behavior is congruent with a person’s values and goals.

#### 2.2.5. Happiness, meaning, and balance

Emotion serves as an embodied reaction that informs a person regarding their state of consistency (Clore and Palmer, 2009). As cognitive processes are typically outside a person’s awareness (Wegner, 2002), heightened emotion, whether negative or positive, provides information regarding the way an individual perceives their experience as personally relevant and meaningful or not (Clore and Schnall, 2005). Studies show that meaning generates positive emotions (King et al., 2006), whereas negative emotions signal low levels or absence of meaning (Wong et al., 2021). Negative affect can activate cognitive processes that may result in re-adjustments in the internal organization of the cognitive patterns (e.g., changes in value priorities) and/or on the behavioral level of functioning (Brandtstädter and Greve, 1994). Such processes aim to maintain consistency between cognitive and physical contexts and to restore emotion (Heady, 2006; Mason et al., 2019) by reducing perceived discrepancies between the individual’s ideal states (e.g., cognitive environment) and actual states (e.g., physical environment). These findings support the hypothesis that emotion may serve as a signal regarding the connection of cognition to behavior through meaning.

In conclusion, emotion provides vital information that enables a person to make adaptive re-adjustments in response to change and maintain psychological balance and well-being. Such dynamic re-adjustments rely on a negative feedback loop mechanism (e.g., Carver and Scheier, 2019) that aims to reduce perceived discrepancies between a person’s cognitive and physical environmental contexts and restore emotion.

## 3. Summary

Altogether, the present article introduces a model that demonstrates that happiness, as the experience of positive emotions, is a marker of well-being, whereas meaning, as ongoing cognitive processes, serves to maintain it. In this sense, happiness indicates the presence of meaning that allows people to make sense of themselves and feel connected to the outside world. Intense negative emotions indicate a state of inconsistency and aim to re-activate meaning and restore emotion. The model generates the hypothesis that meaningful interpretations of perceived discrepancies between a person’s cognitive patterns (e.g., goals) and behavioral patterns (e.g., goal pursuit) may lead to adaptive re-adjustments that restore positive emotion and well-being. This hypothesis may be tested in future longitudinal studies.

### 3.1. Concluding remarks

Although the notion of happiness is expanding incrementally toward including meaning as one of its dimensions, the conceptualization of different types of happiness presents a barrier in understanding the functional psychological abilities that contribute to well-being. Instead of adopting a new term, psychologists may promote clear communication by describing what is measured (e.g., personal expressiveness as a marker of happiness) and by specifying the level of functioning under investigation (i.e., emotion, cognition, behavior).

As it may never be possible to measure everything that is associated with happiness (Huta, 2018), this article proposes moving beyond the concern of *what* makes a good life and instead, focus the research inquiry on the principles that facilitate the experience of happiness. As a step toward this direction, this article draws on existing knowledge and constructs a coherent framework that identifies consistency as the primary prerequisite for happiness, which relies on meaning to translate a state of consistency as positive emotion. Thus, an individual experiences happiness and meaning when their behavior manifests what is mostly important to them. The model of happiness celebrates psychological complexity and attempts to explain the psychological conditions of what it means to *feel good*. Keeping an open enquiry around the underlying mechanism that regulates functionality may lead to making a better sense of the overall human experience. Investigating the processes that underlie this kind of complex psychological phenomena can facilitate research progress and collaborations from different fields of psychology.

## Author contributions

The author confirms being the sole contributor of this work and has approved it for publication.

## Funding

The author would like to thank the dual career department at ETH Zurich for covering the publication cost of this article.

## Conflict of interest

The author declares that the research was conducted in the absence of any commercial or financial relationships that could be construed as a potential conflict of interest.

## Publisher’s note

All claims expressed in this article are solely those of the authors and do not necessarily represent those of their affiliated organizations, or those of the publisher, the editors and the reviewers. Any product that may be evaluated in this article, or claim that may be made by its manufacturer, is not guaranteed or endorsed by the publisher.

## References

[ref2] Alexander R. Aragón O. R. Bookwala J. Cherbuin N. Gatt J. M. Kahrilas I. J.. (2021). The neuroscience of positive emotions and affect: implications for cultivating happiness and wellbeing. Neurosci. Biobehav. Rev. 121, 220–249. doi: 10.1016/j.neubiorev.2020.12.002, PMID: 33307046

[ref3] Allemand M. Hill P. L. Ghaemmaghami P. Martin M. (2012). Forgivingness and subjective well-being in adulthood: the moderating role of future time perspective. J. Res. Pers. 46, 32–39. doi: 10.1016/j.jrp.2011.11.004

[ref4] Arnold V. I. Afrajmovich V. S. Il’yashenko Y. S. Shil’nikov L. P. (2013). Dynamical systems V: Bifurcation theory and catastrophe theory (5). Berlin: Springer Science & Business Media.

[ref5] Bai J. Mo K. Peng Y. Hao W. Qu Y. Lei X.. (2021). The relationship between the use of mobile social media and subjective well-being: the mediating effect of boredom proneness. Front. Psychol. 11:568492. doi: 10.3389/fpsyg.2020.568492, PMID: 33584406 PMC7874194

[ref6] Bardi A. Lee J. A. Hofmann-Towfigh N. Soutar G. (2009). The structure of intraindividual value change. J. Pers. Soc. Psychol. 97, 913–929. doi: 10.1037/a0016617, PMID: 19857010

[ref7] Besika A. (2022). A within-study cross-validation of the values-as-ideals measure: levels of value orientation explain variability in well-being. Heliyon 8:e12131. doi: 10.1016/j.heliyon.2022.e12131, PMID: 36582713 PMC9792749

[ref8] Besika A. Horn A. B. Martin M. (2021). Psychological balance scale: validation studies of an integrative measure of well-being. Front. Psychol. 12, 1–17. doi: 10.3389/fpsyg.2021.727737, PMID: 34603149 PMC8483246

[ref9] Besika A. Schooler J. W. Verplanken B. Mrazek A. J. Ihm E. D. (2022). A relationship that makes life worth living: levels of value orientation explain differences in meaning and life satisfaction. Heliyon 8:e08802. doi: 10.1016/j.heliyon.2022.e08802, PMID: 35146155 PMC8802095

[ref10] Bonebright C. A. Clay D. L. Ankenmann R. D. (2000). The relationship of workaholism with work–life conflict, life satisfaction, and purpose in life. J. Couns. Psychol. 47, 469–477. doi: 10.1037/0022-0167.47.4.469

[ref11] Brandtstädter J. Greve W. (1994). The aging self: stabilizing and protective processes. Dev. Rev. 14, 52–80. doi: 10.1006/drev.1994.1003

[ref12] Cacioppo S. Bianchi-Demicheli F. Frum C. Pfaus J. G. Lewis J. W. (2012). The common neural bases between sexual desire and love: a multilevel kernel density fMRI analysis. J. Sex. Med. 9, 1048–1054. doi: 10.1111/j.1743-6109.2012.02651.x, PMID: 22353205

[ref13] Carver C. S. Scheier M. F. (2019). “A self-regulatory viewpoint on human behavior” in The Oxford handbook of human motivation. ed. Ryan R. M. (Oxford: Oxford University Press), 27–46.

[ref14] Caspi A. McClay J. Moffitt T. E. Mill J. Martin J. Craig I. W.. (2002). Role of genotype in the cycle of violence in maltreated children. Science 297, 851–854. doi: 10.1126/science.1072290, PMID: 12161658

[ref15] Cieciuch J. Davidov E. Algesheimer R. (2016). The stability and change of value structure and priorities in childhood: a longitudinal study. Soc. Dev. 25, 503–527. doi: 10.1111/sode.12147

[ref16] Clore G. L. Ortony A. (2008). “Appraisal theories: how cognition shapes affect into emotion” in Handbook of Emotions. eds. Lewis M. Haviland-Jones J. M. Barrett L. F. (New York: The Guilford Press), 628–642.

[ref17] Clore G. L. Palmer J. (2009). Affective guidance of intelligent agents: how emotion controls cognition. Cogn. Syst. Res. 10, 21–30. doi: 10.1016/j.cogsys.2008.03.002, PMID: 19255620 PMC2599948

[ref18] Clore G. L. Schnall S. (2005). “The influence of effect on attitude” in Handbook of attitudes and attitude change: Basic principles. eds. Johnson B. T. Albarracın D. Zanna M. P. (Mahwah: Lawrence Erlbaum Associates Publishers), 437–490.

[ref19] Coelho G. L. D. H. Hanel P. H. Johansen M. K. Maio G. R. (2019). Mapping the structure of human values through conceptual representations. Eur. J. Personal. 33, 34–51. doi: 10.1002/per.2170

[ref20] Cowan H. R. (2019). Can a good life be unsatisfying? Within-person dynamics of life satisfaction and psychological well-being in late midlife. Psychol. Sci. 30, 697–710. doi: 10.1177/0956797619831981, PMID: 30897028

[ref21] Cowen E. L. (1991). In pursuit of wellness. Am. Psychol. 46, 404–408. doi: 10.1037/0003-066X.46.4.404

[ref22] Csabonyi M. Phillips L. J. (2020). Meaning in life and substance use. J. Humanist. Psychol. 60, 3–19. doi: 10.1177/0022167816687674

[ref23] Csikszentmihalyi M. (1990). Flow. New York: Harper Collins Publishers Ltd.

[ref24] Cummins R. A. (2003). Normative life satisfaction: measurement issues and a homeostatic model. Soc. Indic. Res. 64, 225–256. doi: 10.1023/A:1024712527648

[ref25] Davis K. L. Panksepp J. (2011). The brain’s emotional foundations of human personality and the affective neuroscience personality scales. Neurosci. Biobehav. Rev. 35, 1946–1958. doi: 10.1016/j.neubiorev.2011.04.004 21527289

[ref27] Diener E. (2009). Assessing subjective well-being: Progress and opportunities. Assess. WellBeing 39, 25–66. doi: 10.1007/978-90-481-2354-4_3

[ref28] Diener E. Diener C. (1996). Most people are happy. Psychol. Sci. 7, 181–185. doi: 10.1111/j.1467-9280.1996.tb00354.x

[ref01] Dressler W. W. Bindon J. (2000). The health consequences of cultural consonance: Cultural dimensions of lifestyle, social support, and arterial blood pressure in an African American community. American Anthropologist 102, 244–260.

[ref29] Feng L. Zhong H. (2021). Interrelationships and methods for improving university students' sense of gain, sense of security, and happiness. Front. Psychol. 12:729400. doi: 10.3389/fpsyg.2021.729400, PMID: 34630241 PMC8497965

[ref30] Ford D. N. (1999). A behavioral approach to feedback loop dominance analysis. Syst. Dyn. Rev. 15, 3–36. doi: 10.1002/(SICI)1099-1727(199921)15:1<3::AID-SDR159>3.0.CO;2-P

[ref31] Gaertner L. Sedikides C. O’Mara E. M. (2008). On the motivational primacy of the individual self: ‘I’ is stronger than ‘we.’. Soc. Personal. Psychol. Compass 2, 1913–1929. doi: 10.1111/j.1751-9004.2008.00142.x

[ref32] George L. S. Park C. L. (2017). The multidimensional existential meaning scale: a tripartite approach to measuring meaning in life. J. Posit. Psychol. 12, 613–627. doi: 10.1080/17439760.2016.1209546

[ref33] Grant A. M. Schwartz B. (2011). Too much of a good thing: the challenge and opportunity of the inverted U. Perspect. Psychol. Sci. 6, 61–76. doi: 10.1177/1745691610393523 26162116

[ref34] Heady B. (2006). Discussion papers Bruce Headey happiness: revising set point theory and dynamic equilibrium theory to account for long term change, (no 607). DIW Discussion Papers. Available at: https://www.diw.de/documents/publikationen/73/diw_01.c.44536.de/dp607.pdf

[ref35] Heady B. Wearing A. J. (1992). A Theory of Subjective Well-Being. Melbourne: Longman Cheshire.

[ref36] Heine S. J. Proulx T. Vohs K. D. (2006). The meaning maintenance model: on the coherence of social motivations. Personal. Soc. Psychol. Rev. 10, 88–110. doi: 10.1207/s15327957pspr1002_1, PMID: 16768649

[ref37] Heintzelman S. J. King L. A. (2014). (the feeling of) meaning-as-information. Personal. Soc. Psychol. Rev. 18, 153–167. doi: 10.1037/a0035049, PMID: 24501092

[ref38] Hernandez R. Bassett S. M. Boughton S. W. Schuette S. A. Shiu E. W. Moskowitz J. T. (2018). Psychological well-being and physical health: associations, mechanisms, and future directions. Emot. Rev. 10, 18–29. doi: 10.1177/175407391769782, PMID: 36650890 PMC9841922

[ref39] Higgins E. T. (1987). Self-discrepancy: a theory relating self and affect. Psychol. Rev. 94, 319–340. doi: 10.1037/0033-295X.94.3.319, PMID: 3615707

[ref40] Hindriks F. Douven I. (2018). Nozick’s experience machine: An empirical study. Philos. Psychol. 31, 278–298. doi: 10.1080/09515089.2017.1406600

[ref41] Horak F. B. (2006). Postural orientation and equilibrium: what do we need to know about neural control of balance to prevent falls? Age Ageing 35, ii7–ii11. doi: 10.1093/ageing/afl077 16926210

[ref42] Hornsey M. J. Bain P. G. Harris E. A. Lebedeva N. Kashima E. S. Guan Y.. (2018). How much is enough in a perfect world? Cultural variation in ideal levels of happiness, pleasure, freedom, health, self-esteem, longevity, and intelligence. Psychol. Sci. 29, 1393–1404. doi: 10.1177/0956797618768058, PMID: 29889603

[ref43] Huta V. (2018). Eudaimonia versus Hedonia: what is the difference? And is it real. Int. J. Exist. Psychol. Psychother. 7:8.

[ref44] Huta V. (2020). How distinct are eudaimonia and hedonia? It depends on how they are measured. J. Well Being Assess. 4, 511–537. doi: 10.1007/s41543-021-00046-4

[ref45] Jamieson J. P. Crum A. J. Goyer J. P. Marotta M. E. Akinola M. (2018). Optimizing stress responses with reappraisal and mindset interventions: an integrated model. Anxiety Stress Cop. 31, 245–261. doi: 10.1080/10615806.2018.1442615, PMID: 29471669

[ref02] Jung C. G. (1959). Face to face with Carl Gustav Jung by John Freeman. [YouTube]. Available at: https://www.youtube.com/watch?v=2AMu-G51yTY

[ref46] Jung C. G. (1977). “CG Jung speaking: interviews and encounters” in The structure and dynamics of the psyche, CW 8. eds. McGuire W. Hull R. F. C. (Princeton: Princeton University Press).

[ref47] Karataş Z. Uzun K. Tagay Ö. (2021). Relationships between the life satisfaction, meaning in life, hope and COVID-19 fear for Turkish adults during the COVID-19 outbreak. Front. Psychol. 12:633384. doi: 10.3389/fpsyg.2021.633384, PMID: 33776856 PMC7990893

[ref48] Kashdan T. B. Biswas-Diener R. King L. A. (2008). Reconsidering happiness: the costs of distinguishing between hedonics and eudaimonia. J. Posit. Psychol. 3, 219–233. doi: 10.1080/17439760802303044

[ref49] King L. A. Hicks J. A. (2021). The science of meaning in life. Annu. Rev. Psychol. 72, 561–584. doi: 10.1146/annurev-psych-072420-122921 32898466

[ref50] King L. A. Hicks J. Del Gaiso A. K. (2006). Positive affect and the experience of meaning in life. J. Pers. Soc. Psychol. 90, 179–196. doi: 10.1037/0022-3514.90.1.179 16448317

[ref51] Kringelbach L. Berridge K. C. (2010). The functional neuroanatomy of pleasure and happiness. Discov. Med. 9, 579–587. 20587348 PMC3008353

[ref52] Kwon Y. J. Park S. J. Jefferson J. Kim K. (2013). The effect of open and closed kinetic chain exercises on dynamic balance ability of normal healthy adults. J. Phys. Ther. Sci. 25, 671–674. doi: 10.1589/jpts.25.671, PMID: 24259825 PMC3805008

[ref53] Lew B. Chistopolskaya K. Osman A. Huen J. M. Y. Abu Talib M. Leung A. N. M. (2020). Meaning in life as a protective factor against suicidal tendencies in Chinese university students. BMC Psychiatry 20, 1–9. doi: 10.1186/s12888-020-02485-4 32070298 PMC7027298

[ref54] Lomas T. (2021). Life balance and harmony: Wellbeing's golden thread. Int. J. Wellbeing 11, 50–68. doi: 10.5502/ijw.v11i1.1477

[ref55] Lomas T. VanderWeele T. J. (2023). The complex creation of happiness: multidimensional conditionality in the drivers of happy people and societies. J. Posit. Psychol. 18, 15–33. doi: 10.1080/17439760.2021.1991453

[ref56] MacLean P. (1978). “A mind of three minds: educating the triune brain” in Education and the brain, 77th yearbook of the National Society for the study of education. ed. JS M. A. C. (Chicago: University of Chicago Press), 308–342.

[ref57] Marco J. H. Cañabate M. Llorca G. Pérez S. (2020). Meaning in life moderates hopelessness, suicide ideation, and borderline psychopathology in participants with eating disorders: a longitudinal study. Clin. Psychol. Psychother. 27, 146–158. doi: 10.1002/cpp.2414, PMID: 31765024

[ref58] Marks S. R. MacDermid S. M. (2006). Multiple roles and the self: a theory of role balance. J. Marriage Fam. 58:417. doi: 10.2307/353506

[ref59] Mason T. B. Smith K. E. Engwall A. Lass A. Mead M. Sorby M.. (2019). Self-discrepancy theory as a transdiagnostic framework: a meta-analysis of self-discrepancy and psychopathology. Psychol. Bull. 145, 372–389. doi: 10.1037/bul0000186, PMID: 30640499

[ref60] McCourt K. Bouchard T. J. Jr. Lykken D. T. Tellegen A. Keyes M. (1999). Authoritarianism revisited: genetic and environmental influences examined in twins reared apart and together. Personal. Individ. Differ. 27, 985–1014. doi: 10.1016/S0191-8869(99)00048-3

[ref61] Nozick R. (1974). Anarchy, state, and utopia. New York: Basic Books.

[ref62] Oleś P. Jankowski T. (2018). Positive orientation—a common base for hedonistic and eudemonistic happiness? Appl. Res. Qual. Life 13, 105–117. doi: 10.1007/s11482-017-9508-9, PMID: 29492164 PMC5813070

[ref63] Panksepp J. (2003). At the interface of the affective, behavioral, and cognitive neurosciences: decoding the emotional feelings of the brain. Brain Cogn. 52, 4–14. doi: 10.1016/S0278-2626(03)00003-4, PMID: 12812799

[ref64] Panksepp J. (2005). Affective consciousness: Core emotional feelings in animals and humans. Conscious. Cogn. 14, 30–80. doi: 10.1016/j.concog.2004.10.004, PMID: 15766890

[ref65] Panksepp J. Watt D. (2011). What is basic about basic emotions? Lasting lessons from affective neuroscience. Emot. Rev. 3, 387–396. doi: 10.1177/1754073911410741

[ref66] Perone S. Simmering V. R. Buss A. T. (2021). A dynamical reconceptualization of executive-function development. Perspect. Psychol. Sci. 16, 1198–1208. doi: 10.1177/1745691620966792, PMID: 33593126 PMC8364921

[ref67] Petrounias Ε. (2018). Dictionary of standard modern Greek: Dictionary of common Neohellenic. Thessaloniki: Manolis Triantafyllidis Foundation.

[ref68] Reker G. T. Wong P. T. P. (1988). “Aging as an individual process: toward a theory of personal meaning” in Emergent theories of aging. eds. Birren J. E. Bengtson V. L. (New York: Springer Publishing Company), 214–246.

[ref69] Rokeach M. (1973). The nature of human values. Mumbai: Free press.

[ref70] Rose H. Womick J. King L. A. (2023). Purpose maintained: adverse childhood experiences and meaning in life. J. Pers. doi: 10.1111/jopy.12820, PMID: 36748110

[ref71] Ross D. (1956). Aristotle: the Nicomachean ethics. Philosophy 31:77.

[ref72] Rushworth M. F. Noonan M. P. Boorman E. D. Walton M. E. Behrens T. E. (2011). Frontal cortex and reward-guided learning and decision-making. Neuron 70, 1054–1069. doi: 10.1016/j.neuron.2011.05.014 21689594

[ref73] Ryff C. D. Lee C. Keyes M. (1995). The structure of psychological well-being revisited. J. Pers. Soc. Psychol. 69, 719–727. doi: 10.1037/0022-3514.69.4.719 7473027

[ref74] Sanbonmatsu D. M. Johnston W. A. (2019). Redefining science: the impact of complexity on theory development in social and behavioral research. Perspect. Psychol. Sci. 14, 672–690. doi: 10.1177/1745691619848688, PMID: 31185185

[ref75] Schimmack U. (2008). “The structure of subjective well-being” in The science of subjective well-being. eds. Eid M. Larsen R. J. (New York: Guildford Press), 97–123.

[ref76] Schimmel J. (2013). “Development as happiness: the subjective perception of happiness and UNDP’s analysis of poverty, wealth and development” in The exploration of happiness. ed. Delle Fave A. (London: Springer)

[ref77] Schnell T. Krampe H. (2020). Meaning in life and self-control buffer stress in times of COVID-19: moderating and mediating effects with regard to mental distress. Front. Psychol. 11:e582352. doi: 10.3389/fpsyt.2020.582352 PMC7538834 33173525

[ref78] Schöner G. Kelso J. A. S. (1988). Dynamic pattern generation in behavioral and neural systems. Science 239, 1513–1520. doi: 10.1126/sci-ence.3281253 3281253

[ref79] Schwartz S. H. (1992). Universals in the content and structure of values: theoretical advances and empirical tests in 20 countries. Adv. Exp. Soc. Psychol. 25, 1–65. doi: 10.1016/S0065-2601(08)60281-6

[ref80] Schwartz S. H. Cieciuch J. (2021). Measuring the refined theory of individual values in 49 cultural groups: psychometrics of the revised portrait value questionnaire. Assessment 29, 1005–1019. doi: 10.1177/1073191121998760, PMID: 33682477 PMC9131418

[ref81] Seligman M. E. P. (2002). Authentic happiness. Mumbai: Free Press.

[ref82] Sortheix F. M. Schwartz S. H. (2017). Values that underlie and undermine well–being: variability across countries. Eur. J. Personal. 31, 187–201. doi: 10.1002/per.2096

[ref83] Tatarkiewicz W. (1976) Analysis of happiness. Den Haag: Nijhoff.

[ref84] Teasdale N. Lajoie Y. Bard C. Fleury M. Courtemanche R. (1993). “Cognitive processes involved for maintaining postural stability while standing and walking” in Sensorimotor impairment in the elderly (London: Springer), 157–168.

[ref85] Trzebiński J. Cabański M. Czarnecka J. Z. (2020). Reaction to the COVID-19 pandemic: the influence of meaning in life, life satisfaction, and assumptions on world orderliness and positivity. J. Loss Trauma 25, 544–557. doi: 10.1080/15325024.2020.1765098

[ref86] Vallacher R. R. Wegner D. M. (1987). What do people think they’re doing? Action identification and human behavior. Psychol. Rev. 94, 3–15. doi: 10.1037/0033-295X.94.1.3

[ref1] Vittersø J. (2016). Handbook of eudaimonic well-being. London: Springer International Publishing.

[ref87] Waterman A. S. (2022). Toward a theory of maldaimonia. J. Theor. Philos. Psychol. 42, 202–219. doi: 10.1037/teo0000198

[ref88] Wegner D. M. (2002). The illusion of conscious will MIT Press.

[ref89] Wolfram H. J. (2022). Meaning in life, life role importance, life strain, and life satisfaction. Curr. Psychol. 1–13. doi: 10.1007/s12144-022-04031-9

[ref90] Wong P. T. Arslan G. Bowers V. L. Peacock E. J. Kjell O. N. E. Ivtzan I.. (2021). Self-transcendence as a buffer against COVID-19 suffering: the development and validation of the self-transcendence measure-B. Front. Psychol. 12:648549. doi: 10.3389/fpsyg.2021.648549, PMID: 34690853 PMC8527188

[ref91] World Health Organization (2021). Geneva Chapter for Well-Being. Geneva: World Health Organization.

[ref92] Zittoun T. Brinkmann S. (2012). “Learning as meaning making” in Encyclopedia of the sciences of learning. ed. Steel N. M., 1809–1811.

